# Haloperidol-Induced Neuroleptic Malignant Syndrome: A Case Report

**DOI:** 10.7759/cureus.57276

**Published:** 2024-03-30

**Authors:** Jordan P Fader, Madison Walker Evans, Austin M Lundgren, Trenton B Sigmund, Taylor J Allen

**Affiliations:** 1 Medicine, College of Osteopathic Medicine, Kansas City University, Kansas City, USA; 2 Medicine, College of Osteopathic Medicine, A.T. Still University Kirksville, Kirksville, USA; 3 Medicine, Capital Region Medical Center, University of Missouri Health Care, Jefferson, USA

**Keywords:** neuroleptic agents, antipsychotics, haloperidol, nms, neuroleptic malignant syndrome

## Abstract

Neuroleptic malignant syndrome (NMS) is a severe reaction to antipsychotic medications characterized by fever, muscle rigidity, altered mental status, and autonomic dysfunction. Here, we describe the case of a 58-year-old female who presented with altered mental status two days after open reduction and internal fixation of the hip. A rapid response team was called when the patient appeared agitated with increased respiratory demand. After being intubated and moved to the ICU, she became febrile and rigid. A preliminary diagnosis of metabolic encephalopathy of unknown origin was made. Before being transported to the ICU, the patient was given multiple haloperidol doses in addition to her continued at-home medication, paroxetine, for major depressive disorder. The differential diagnosis included a workup for NMS, serotonin syndrome, and infectious processes. Once NMS was determined as the most likely etiology, all antipsychotic and serotonergic medications were discontinued. Then dantrolene and amantadine were administered, which resulted in clinically significant improvement. This case report demonstrates the importance of early identification of and intervention for NMS.

## Introduction

Neuroleptic malignant syndrome (NMS) is a severe reaction to antipsychotic medications and dopamine antagonists or because of a fast withdrawal of dopaminergic medications [1]. Antipsychotic medications, such as haloperidol, act as dopamine receptor antagonists; hence, they effectively reduce the transmission of dopaminergic neuronal signals [1]. NMS requires fast identification and treatment to reduce the chances of morbidity and mortality [1]. Although there is not one universally accepted set of diagnostic criteria for NMS, The Diagnostic and Statistical Manual of Mental Disorders, Fifth Edition, Text Revision (DSM-5-TR) lays forward one set of criteria [2,3]. Symptoms can vary between cases but can include hyperthermia greater than 38 degrees Celsius on two separate occasions, diaphoresis, rigidity unresponsive to antiparkinsonian drugs, creatine kinase four times the upper limit of normal, tachycardia, tachypnea, hypertension, urinary incontinence, altered mental status, and pallor [3]. Before an NMS diagnosis is made, the workup should rule out infection, toxic and metabolic causes, and neuropsychiatric conditions [3]. Laboratory evaluation may reveal leukocytosis, hypoxia, metabolic acidosis, decreased serum iron levels, elevated concentrations of muscle breakdown products, and catecholamines, but these are not all necessary to make the diagnosis of, and are not specific for, NMS [3].

This case was previously presented as a poster at the Missouri Society of the American College of Osteopathic Family Physicians Winter Family Medicine Update meeting on January 20, 2024.

## Case presentation

Here, we describe the case of a 58-year-old female with a past medical history of coronary artery disease, heart failure with reduced ejection fraction, chronic obstructive pulmonary disease, and major depressive disorder who underwent an open reduction with internal fixation procedure for a hip fracture. On the second postoperative day, she showed signs of agitation and was therefore prescribed 1 mg of haloperidol to be provided intravenously on a scheduled basis every four hours. She was simultaneously prescribed 1 mg of lorazepam to be given as needed. After administration of eleven 1 mg haloperidol doses, the patient remained confused and agitated. At this time, the admitting physician prescribed 5 mg haloperidol every four hours. She was given two doses, and around the time the second dose was administered, a rapid response team was called as the patient was unresponsive and had labored breathing. The patient was transferred to the ICU on noninvasive ventilatory support. She then became tachycardic at 152 beats per minute and was intubated because of increasing respiratory demand. Haloperidol, lorazepam, and opioid pain medications were discontinued at the time of ICU transfer. A subsequent chest X-ray confirmed appropriate endotracheal tube placement and was otherwise unremarkable. MRI could not be performed because of the patient’s previously implanted spinal cord stimulator. Computed tomography of the head was thus performed and was unremarkable. A D-dimer was mildly elevated, which is expected after a surgical procedure. The diagnosis at that time was metabolic encephalopathy of unknown origin.

**Figure 1:**
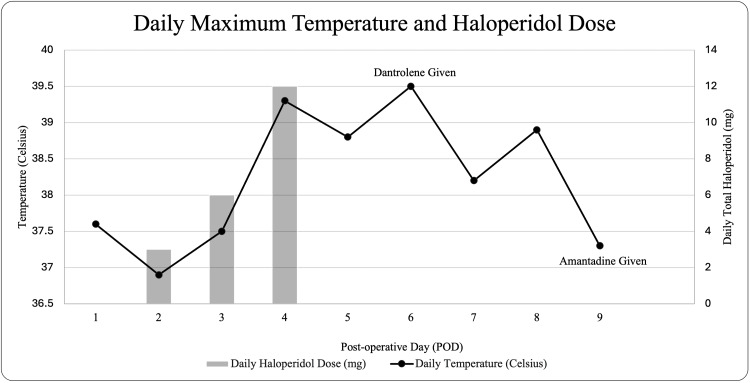
The patient's daily maximum body temperature and daily total haloperidol dosing by postoperative day

On the fourth postoperative day, the patient developed a fever of 39.3 degrees Celsius. The ICU physician back-ordered a serum creatine kinase level and initiated external cooling measures. The differential diagnosis at this time included delirium tremens, hydromorphone reaction, infection, serotonin syndrome, and NMS. However, the patient’s family reported no alcohol consumption or previous hydromorphone reactions. Additionally, there was no clear source of infection; urine and blood cultures were negative, and a lumbar puncture was unremarkable. Although the symptoms did not quite fit the clinical picture for serotonin syndrome, the patient’s home-daily 10 mg paroxetine was discontinued, and a trial of 12 mg cyproheptadine was administered on postoperative day five. When the patient’s fever and rigidity persisted on the sixth day postsurgery, and the creatine kinase was elevated at 542 U/L (normal range 34-135 U/L for females), 85 mg of dantrolene was administered [4]. While the patient's maximum temperature improved, clinical signs such as marked rigidity persisted until postoperative day nine, when the patient was given 100 mg amantadine. After the administration of amantadine, the patient remained afebrile and began to show signs of clinical improvement. A timeline of these events is displayed in Figure 1. 

This case presents a mild to moderate case of NMS that was recognized early with prompt administration of dantrolene and amantadine. Once the patient was extubated, she was profoundly weak. The patient spent eight days in inpatient rehabilitation after hospital discharge and then began performing home health physical therapy to help with postoperative recovery and regaining strength lost during a period of immobility. On follow-up with primary care and orthopedics, the patient did not note any lingering effects, and no residual signs of NMS were present. She continues to improve and restarted her home paroxetine at 10 mg per day without issue.

The path to NMS diagnosis was complicated in this case as the patient was sedated and intubated. These efforts were necessary to stabilize the patient, but it limited the collection of additional history. Moreover, NMS is a diagnosis of exclusion, so the differential diagnoses discussed above had to first be ruled out. While no diagnostic criteria have been established for diagnosing NMS, there are multiple clinical findings and lab values that can aid in diagnosis. In this case, exposure to a dopamine antagonist, fever, rigidity, altered mental status, and elevated creatine kinase help aid in diagnosing NMS [2].

## Discussion

Haloperidol is a first-generation antipsychotic that exerts its effects by blocking dopamine D2 receptors in the brain [5]. It is approved for use in schizophrenia, Tourette’s syndrome, severe behavioral disorders in children, and hyperactivity in children as well as off-label for the treatment of acute agitation [5]. Patients who have adverse reactions to haloperidol and develop NMS often present with rigidity, hyperthermia, and altered mental status [1]. Common treatments for NMS include bromocriptine mesylate, a dopamine agonist, and dantrolene sodium, a muscle relaxant that blocks calcium release in the muscle [1]. Additional treatment options include amantadine and other agents that work to increase the levels of dopamine in the brain [1]. Any potential contributing psychotropic agents, including serotonergic drugs, should be discontinued if NMS is suspected [1].

Both the diagnosis and treatment of NMS in this patient demonstrate an excellent case of interdisciplinary medical care but also highlight the importance of judicious prescription of neuroleptic agents. This patient was not on long-term antipsychotic medication before being prescribed 1 mg haloperidol scheduled on a four-hour basis. Her dose was later increased to 5 mg every four hours, and she received this dose twice. It is not clear to the authors why this was done, as our experience with the patient began at the time of ICU admission following the rapid response. While patients can be prescribed larger quantities of haloperidol and not have symptoms, it is best if haloperidol-naive patients are prescribed low doses (0.5 mg to 1 mg) with a maximum daily dose of 5 mg [6]. Moreover, it is recommended that haloperidol be administered on an as-needed basis and not given in a scheduled, prophylactic manner [6]. A more conservative approach to agitation for postsurgical patients may include the use of newer and better-tolerated second-generation (atypical) antipsychotics, benzodiazepines, or dexmedetomidine [2]. Furthermore, it should be noted that, although NMS most often occurs insidiously during the first one to two weeks of acute intervention with neuroleptics, it can occur after a single dose of antipsychotic medication or after treatment with the same agent, at the same dose, for many years [7]. While not a dose-dependent phenomenon, higher doses, recent or rapid dose escalation, and parenteral administration are risk factors for NMS [8].

## Conclusions

The administration of haloperidol for acute onset agitation within the hospital setting is a viable treatment option. However, the medication comes with substantial risks that should be considered before administration. Additionally, all members of the medical team should be aware of when medications with potentially severe side effects are prescribed so that multiple people are watching for such side effects. The team also needs to be ready to discontinue medications and quickly change the course of treatment when adverse side effects begin to emerge. When drugs causing side effects are swiftly stopped, patients can go on to have a relatively mild recovery with no long-standing effects from the medications.
